# Avian haemosporidian parasites in captive and free-ranging, wild birds from zoological institutions in Switzerland: Molecular characterization and clinical importance

**DOI:** 10.1016/j.ijppaw.2022.12.005

**Published:** 2022-12-26

**Authors:** Seraina L. Meister, Fabia Wyss, Christian Wenker, Stefan Hoby, Walter U. Basso

**Affiliations:** a Institute of Parasitology, Department of Infectious Diseases and Pathobiology, Vetsuisse Faculty, University of Bern, Länggassstrasse 122, CH-3012, Bern, Switzerland; b Institute of Animal Pathology, Department of Infectious Diseases and Pathobiology, Vetsuisse Faculty, University of Bern, Länggassstrasse 122, CH-3012, Bern, Switzerland; c Zoo Basel, Binningerstrasse 40, CH-4054, Basel, Switzerland; d Berne Animal Park, Tierparkweg 1, CH-3005, Bern, Switzerland

**Keywords:** Avian malaria, Avian haemosporidia, *Plasmodium*, *Haemoproteus*, Birds, PCR

## Abstract

Avian haemosporidian parasites are widespread and infect birds from a broad variety of avian families with diverse consequences ranging from subclinical infections to severe and fatal disease. This study aimed to determine the occurrence and diversity of avian haemosporidia including associated clinical signs and pathomorphological lesions in captive and free-ranging, wild birds from two zoos and the near environment in Switzerland. Blood samples from 475 birds, including 230 captive and 245 free-ranging, wild individuals belonging to 42 different avian species from 15 orders were examined for the presence of avian haemosporidian DNA by a one-step multiplex PCR designed to simultaneously detect and discriminate the genera *Plasmodium*, *Haemoproteus* and *Leucocytozoon* by targeting mitochondrial genome sequences. Positive samples were additionally tested using a nested PCR targeting the cytochrome *b* gene of *Plasmodium* and *Haemoproteus*. The obtained amplicons were bidirectionally sequenced. This study revealed haemosporidian DNA in 42 samples, belonging to ten host species. The most commonly detected lineage was *Plasmodium relictum* SGS1, which was identified in 29 birds (*Phoenicopterus roseus*: n = 24, *Alectoris graeca*: n = 1, *Lamprotornis superbus*: n = 1, *Somateria mollissima*: n = 1, *Spheniscus demersus*: n = 1, *Tetrao urogallus crassirostris*: n = 1), followed by *Haemoproteus* sp. STRURA03 in six avian hosts (*Bubo bubo*: n = 5, *Bubo scandiacus* = 1), *Plasmodium relictum* GRW11 in four individuals (*Phoenicopterus roseus*: n = 3, *Spheniscus demersus*: n = 1) and *Plasmodium elongatum* GRW06 in one *Alectura lathami lathami*. A *Phalacrocorax carbo* was infected with *Plasmodium relictum*, but the exact lineage could not be determined. One mixed infection with *P. relictum* and *Haemoproteus* sp. was detected in a *Bubo scandiacus*. Only five individuals (*Spheniscus demersus*: n = 2, *Somateria mollissima*: n = 1, *Bubo scandiacus*: n = 1, *Alectoris graeca*: n = 1) showed clinical and pathomorphological evidence of a haemosporidian infection.

## Highlights

• Avian heamosporidian parasites were detected in captive birds in Switzerland.
• *Plasmodium relictum*, *P. elongatum* and *Haemoproteus* were found in 10 bird species.
• Subclinical and clinical infections were recorded.
• Genus Spheniscus, Somateria, Bubo and Alectoris showed clinical avian malaria.
• *Leucocytozoon* was not detected in any of the blood samples.

## Graphical abstract

**Figure:**
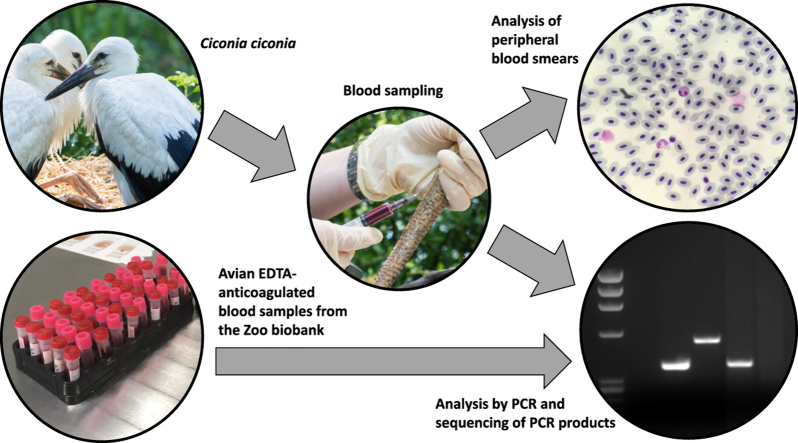

## 1 Introduction

Avian haemosporidiosis is a vector-borne infection, caused by ecologically successful protozoan parasites with an almost cosmopolitan distribution (Valkiūnas, 2005). Avian hematozoa of the genera *Plasmodium*, *Haemoproteus* and *Leucocytozoon* have been detected on every continent except Antarctica, where appropriate dipteran vectors (i.e., Culicidae for *Plasmodium*, Ceratopogonidae and Hippoboscidae for *Haemoproteus*, Simuliidae and Ceratopogonidae for *Leucocytozoon*) are absent (Valkiūnas, 2005). To complete their life cycle, the parasites exploit invertebrate hosts for sexual reproduction and vertebrate hosts for asexual multiplication including exo-erythrocytic and erythrocytic cycles. Depending on the infected avian species, age and immunity of the bird as well as the parasite species and lineage, haemosporidian infection can range from asymptomatic to severe disease with potentially fatal outcome (Palinauskas et al., 2008, 2009, 2011, 2016) resulting in the decline of wild bird populations or in some outbreaks even contributing to the extinction of avian species (Alley et al., 2008; Atkinson and Samuel, 2010; Cannell et al., 2013; Levin et al., 2013; Niebuhr et al., 2016). Based on the immature immune system, juvenile birds are considered to be at highest risk of developing serious disease (Grilo et al., 2016). Also, immunologically naïve animals which had been geographically isolated from these parasites and its vectors, can be highly susceptible to infection with severe clinical signs due to the lack of co-evolution (Atkinson, 2005). Individuals that survive the acute phase become chronically infected and may act as reservoirs for infection of vectors and subsequently of susceptible birds. Analyses of the haemosporidian parasite fauna in wild birds are crucial to determine and understand the introduction of blood parasites to resident host population by migratory avian species (Ricklefs et al., 2017). Migratory birds usually harbor high parasite burdens and a biologically diverse range of avian haemosporidian species (Smith and Ramey, 2015; Chaisi et al., 2019). Although native bird populations do not appear to be severely harmed by the hematozoa, they can act as a source of infection for non-migratory and captive avian species (Bueno et al., 2010; Yoshimura et al., 2014; Chaisi et al., 2019). The transmission to domestic and endangered birds with fatal outcome is an obvious scenario in zoological environments due to the artificial community of organisms, and cases of severe haemosporidiosis were reported in many zoos in Europe, Africa, Asia, South and North America (Fix et al., 1988; Graczyk et al., 1994a; Griner and Sheridan, 1967; Belo et al., 2009; Baron et al., 2014; Vanstreels et al., 2014; Martínez-de la Puente et al., 2015; Chagas et al., 2016), including fatal avian malaria in Atlantic puffins (*Fratercula arctica*) at the Berne Animal Park (Meister et al., 2021) and in African penguins at the Zoo Basel (Cereghetti, 2012) in Switzerland. Nonetheless, the occurrence and prevalence of avian haemosporidian parasites in zoological institutions in Switzerland are only poorly understood.

White storks (*Ciconia ciconia*) are large, long-distance migratory birds belonging to the Swiss native avifauna. Around 50 free-ranging, wild white storks breed at the Zoo Basel every year (Meister et al., 2022). Although various parasite species have been detected in free-ranging white storks in Europe, including trematodes, cestodes, nematodes and protozoa (Franssen et al., 2000; Schuster et al., 2002; Höfle et al., 2003; Andrzejewska et al., 2004; Cabezón et al., 2011; Sitko and Heneberg, 2015; Girisgin et al., 2017; Michalczyk et al., 2020; Meister et al., 2022), scarcely anything is known about haemosporidian infections in these birds.

The aim of this study was to determine the occurrence and diversity of avian haemosporidian parasites in various captive birds from the Berne Animal Park and Zoo Basel as well as in free-ranging, wild avian species with special focus on the white stork population from the Zoo Basel and its near environment using PCR based assays and sequencing of mitochondrial genome sequences, as well as microscopic examination of blood smears from the white storks. In addition, medical records and postmortem reports of the PCR positive animals were analyzed to assess the association of haemosporidian infection with disease. We expected to find different haemosporidian parasites and lineages in this broad range of examined birds and to reveal the clinical significance of haemosporidian infections in infected avian hosts. Furthermore, we aimed to know whether white storks may get infected in Switzerland and serve as carriers of haemosporidia to other geographical regions during migration.

## 2 Materials and methods

### 2.1 Samples

Blood samples were analyzed from 475 birds, representing 15 orders, 19 families and 42 species, of which 230 were captive and 245 were free-ranging, wild individuals (Table 1). From these blood samples, 237 were collected from the *Vena metatarsalis superficialis plantaris* from free-ranging, wild white storks (*Ciconia ciconia*) during the yearly ringing operations in the Swiss cantons Basel-Stadt (Zoo Basel: n = 97, Lange Erlen Animal Park: n = 24, total n = 121), Basel-Landschaft (Allschwil: n = 2, Biel-Benken: n = 6, Binningen: n = 4, Reinach: n = 2, Oberwil: n = 1, total n = 15) and Aargau (Stork Colony Möhlin: n = 78, Kaiseraugst: n = 19, Rheinfelden: n = 4, total n = 101) in 2019 and 2020 (animal experiment permit: 3021/31198). In addition, a total of 238 blood samples stored at −80° Celsius in the biobanks at the Zoo Basel (n = 220, 2001–2021) and Berne Animal Park (n = 18, 2018–2020) were retrospectively analyzed. All these samples were opportunistically collected at the Zoo Basel or Berne Animal Park from avian species belonging to the zoo collection or from injured wild birds that were treated by the corresponding zoo veterinarians in the time periods 2001–2021 and 2018–2020, respectively.

**Table 1:** Occurrence of avian haemosporidian infections in captive and free-ranging, wild (*) avian species from the Zoo Basel and Berne Animal Park detected by multiplex and nested PCR assays. P = *Plasmodium* sp.; H = *Haemoproteus* sp.; L = *Leucocytozoon* sp. N/A = not applicable.

Tested avian species			n	Performed PCR methods						
Order	Family	Species	Total	Multiplex PCR				Nested PCR		Haemosporidian lineage detected by nested PCR
				P	H	L	% infected (CI 95%)	P/H	% infected (CI 95%)	
**Zoo Basel (Basel-Stadt)**			**317**	33	4	0	11.7 (8.4–15.7%)	36	11.4 (8.1–15.4%)	
Anseriformes	Anatidae	Bar-headed goose (*Anser indicus*)	1	0	0	0	0	0	0	N/A
		Barnacle goose (*Branta leucopsis*)	1	0	0	0	0	0	0	N/A
		Black swan (*Cygnus atratus*)	7	0	0	0	0	0	0	N/A
		Common eider (*Somateria mollissima*)	1	1	0	0	100 (2.5–100%)	1	100 (2.5–100%)	*P. relictum* SGS1
		Hooded merganser (*Lophodytes cucullatus*)	1	0	0	0	0	0	0	N/A
		Mute swan (*Cygnus olor*)*	2	0	0	0	0	0	0	N/A
		Ne-ne (*Branta sandvicensis*)	6	0	0	0	0	0	0	N/A
		Red-breasted goose (*Branta ruficollis*)	4	0	0	0	0	0	0	N/A
Bucerotiformes	Bucorvidae	Southern ground hornbill (*Bucorvus leadbeateri*)	4	0	0	0	0	0	0	N/A
Ciconiiformes	Ciconiidae	White stork (*Ciconia ciconia*)*	5 + 97	0	0	0	0	0	0	N/A
Columbiformes	Columbidae	Socorro dove (*Zenaida graysoni*)	2	0	0	0	0	0	0	N/A
Galliformes	Megapodiidae	Australian brush turkey (*Alectura lathami lathami*)	9	1	0	0	11.1 (0.3–48.3%)	1	11.1 (0.3–48.3%)	*P. elongatum* GRW06
	Numididae	Helmeted guineafowl (*Numida meleagris*)	3	0	0	0	0	0	0	N/A
	Phasianidae	Common peafowl (*Pavo cristatus*)	2	0	0	0	0	0	0	N/A
		Crested wood partridge (*Rollulus rouloul*)	1	0	0	0	0	0	0	N/A
		Orpington chicken (*Gallus gallus domesticus*)	1	0	0	0	0	0	0	N/A
Musophagiformes	Musophagidae	Fischer's turaco (*Turaco fischeri*)	1	0	0	0	0	0	0	N/A
Passeriformes	Sturnidae	Hill mynah (*Gracula religiosa*)	1	0	0	0	0	0	0	N/A
		Superb starling (*Lamprotornis superbus*)	1	1	0	0	100 (2.5–100%)	1	100 (2.5–100%)	*P. relictum* SGS1
Pelecaniformes	Pelecanidae	Eastern white pelican (*Pelecanus onocrotalus*)	4	0	0	0	0	0	0	N/A
	Threskiornithidae	Northern bald ibis (*Geronticus eremita*)	7	0	0	0	0	0	0	N/A
		Scarlet ibis (*Eudocimus ruber*)	2	0	0	0	0	0	0	N/A
Phoenicopteriformes	Phoenicopteridae	Greater flamingo (*Phoenicopterus roseus*)	85	27	0	0	31.8 (22.1–42.8%)	27	31.8 (22.1–42.8%)	*P. relictum* SGS1 (n = 24)
										*P. relictum* GRW11 (n = 3)
Psittaciformes	Psittacidae	Blue-crowned parrot (*Loriculus galgulus*)	1	0	0	0	0	0	0	N/A
		Blue-fronted amazon (*Amazona aestiva*)	1	0	0	0	0	0	0	N/A
		Buffon's macaw (*Ara ambiguus*)	1	0	0	0	0	0	0	N/A
Sphenisciformes	Spheniscidae	African penguin (*Spheniscus demersus*)	24	2	0	0	8.3 (1–27%)	2	8.3 (1–27%)	*P. relictum* SGS1 (n = 1)
										*P. relictum* GRW11 (n = 1)
		Gentoo penguin (*Pygoscelis papua*)	7	0	0	0	0	0	0	N/A
		King penguin (*Aptenodytes patagonicus*)	8	0	0	0	0	0	0	N/A
Strigiformes	Strigidae	Eurasian eagle owl (*Bubo bubo*)	5	0	3	0	60 (14.7–94.7%)	3	60 (14.7–94.7%)	*Haemoproteus* sp. STRURA03
		Snowy owl (*Bubo scandiacus*)	1	0	1	0	100 (2.5–100%)	1	100 (2.5–100%)	*Haemoproteus* sp. STRURA03
		Spectacled owl (*Pulsatrix perspicillata*)	4	0	0	0	0	0	0	N/A
		Tawny owl (*Strix aluco*)*	1	0	0	0	0	0	0	N/A
Struthioniformes	Struthionidae	Common ostrich (*Struthio camelus*)	12	0	0	0	0	0	0	N/A
Suliformes	Phalacrocoracidae	Great cormorant (*Phalacrocorax carbo*)	4	1	0	0	25 (0.6–80.6%)	0	0	*P. relictum*
**Lange Erlen Animal Park (Basel-Stadt)**			**24**	0	0	0	0	0	0	
Ciconiiformes	Ciconiidae	White stork (*Ciconia ciconia*)*	24	0	0	0	0	0	0	N/A
**Basel-Land**			**15**	0	0	0	0	0	0	
Ciconiiformes	Ciconiidae	White stork (*Ciconia ciconia*)*	15	0	0	0	0	0	0	N/A
**Aargau**			**101**	0	0	0	0	0	0	
Ciconiiformes	Ciconiidae	White stork (*Ciconia ciconia*)*	101	0	0	0	0	0	0	N/A
**Berne Animal Park**			**18**	3	3	0	33.3 (13.3–59%)	5	27.8 (9.7–53.5%)	
Anseriformes	Anatidae	Diepholzer goose (*Anser anser domestica*)	1	0	0	0	0	0	0	N/A
Charadriiformes	Alcidae	Atlantic puffin (*Fratercula arctica*)	2	0	0	0	0	0	0	N/A
	Recurvirostridae	Pied avocet (*Recurvirostra avosetta*)	1	0	0	0	0	0	0	N/A
Galliformes	Phasianidae	Appenzeller spitzhauben chicken (*Gallus gallus domesticus*)	1	0	0	0	0	0	0	N/A
		Rock partridge (*Alectoris graeca*)	1	1	0	0	100 (2.5–100%)	1	100 (2.5–100%)	*P. relictum* SGS1
		Western capercaillie (*Tetrao urogallus crassirostris*)	1	1	0	0	100 (2.5–100%)	1	100 (2.5–100%)	*P. relictum* SGS1
Pelecaniformes	Pelecanidae	Dalmatian pelican (*Pelecanus crispus*)	5	0	0	0	0	0	0	N/A
Phoenicopteriformes	Phoenicopteridae	Greater flamingo (*Phoenicopterus roseus*)	3	0	0	0	0	0	0	N/A
Strigiformes	Strigidae	Eurasian eagle owl (*Bubo bubo*)	2	0	2	0	100 (15.8–100%)	2	100 (15.8–100%)	*Haemoproteus* sp. STRURA03
		Snowy owl (*Bubo scandiacus*)	1	1	1	0	100 (2.5–100%)	1	100 (2.5–100%)	*Haemoproteus* sp., *P. relictum*

### 2.2 DNA extraction

DNA extraction from the EDTA-anticoagulated blood samples was performed using the DNeasy Blood & Tissue Kit (QIAGEN: Spin-Column Protocol for Animal Blood) according to the manufacturer's protocol for nucleated erythrocytes with the modification that the initial amount of avian blood was increased from 10 to 20 μl. The larger amount of blood aimed the detection of haemosporidian infections in asymptomatic animals with potentially low parasitemia.

### 2.3 One-step multiplex PCR

A one-step multiplex PCR targeting mitochondrial genome sequences for simultaneous detection and discrimination of avian haemosporidian parasites belonging to the genera *Plasmodium*, *Haemoproteus* and *Leucocytozoon* was performed as an initial screening assay as previously described (Ciloglu et al., 2019; Meister et al., 2021). The resulting amplicons were visualized by gel electrophoresis, purified, and bidirectionally sequenced as previously reported (Meister et al., 2021), followed by comparison with sequences on GenBank using the Nucleotide BLAST algorithm (https://blast.ncbi.nlm.nih.gov/Blast.cgi).

### 2.4 Nested PCR

For confirmation and exact identification of the *Plasmodium* and *Haemoproteus* species and lineages detected by the one-step multiplex PCR, a nested PCR protocol targeting the cytochrome *b* (cyt *b*) gene for detection of *Plasmodium* and *Haemoproteus* infections was additionally performed as previously described (Waldenström et al., 2004; Meister et al., 2021). The resulting amplicons were visualized by gel electrophoresis, purified, and bidirectionally sequenced as previously reported (Meister et al., 2021), followed by comparison with sequences on GenBank (http://blast.ncbi.nlm.nih.gov/Blast.cgi) and on the MalAvi database (http://130.235.244.92/Malavi/blast.html) using the Nucleotide BLAST algorithm.

### 2.5 Blood smears

During the yearly ringing operations of the white storks in 2019 and 2020 (n = 237), two blood smears per animal were performed from peripheral blood samples using the wedge technique. The blood smears were air dried, fixated with methanol, stained with Giemsa at room temperature for 40 min, washed, air dried and microscopically analyzed by the first author at 1000× magnification with immersion oil for at least 20 min (Bauer, 2006). For the retrospectively analyzed blood samples from the zoo biobanks, blood smears were unfortunately not available and were not prepared for this study since this should be done at the time of collection due to storage-associated changes, which invariably occur.

## 3 Results

### 3.1 One-step multiplex PCR and sequencing

An infection with *Plasmodium relictum* was diagnosed in 34 of the 475 birds (*Phoenicopterus roseus*: n = 27, *Spheniscus demersus*: n = 2, *Alectoris graeca*: n = 1, *Lamprotornis superbus*: n = 1, *Phalacrocorax carbo*: n = 1, *Somateria mollissima*: n = 1, *Tetrao urogallus crassirostris*: n = 1). All 34 amplified sequences were identical to each other. Additionally, in one *Alectura lathami lathami* individual, an amplicon showing two base differences respect to this sequence was obtained, but BLAST analysis did not allow a discrimination at the *Plasmodium* species level. An infection with *Haemoproteus* sp. was diagnosed in six animals (*Bubo bubo*: n = 5, *Bubo scandiacus*: n = 1). In a sample from a further *Bubo scandiacus*, a mixed infection with *Plasmodium* sp. and *Haemoproteus* sp. was detected. The obtained sequences were identical to *P. relictum* and *Haemoproteus* sp. sequences detected in other individuals in the study, respectively. *Leucocytozoon* spp. was not detected in any of the blood samples. The occurrence of the different avian haemosporidian lineages and their prevalence are summarized in Table 1, Table 2, respectively.

**Table 2:** Prevalence of haemosporidian infections in captive birds from the Zoo Basel and Berne Animal Park.

Detected Haemosporidian parasite	Positive individuals (%; CI 95%) by multiplex PCR			Positive individuals (%; CI 95%) by nested PCR		
	Zoo Basel (n = 211)	Berne Animal Park (n = 18)	TOTAL (n = 229)	Zoo Basel (n = 211)	Berne Animal Park (n = 18)	TOTAL (n = 229)
*Plasmodium*	33 (15.6.%; 11.0–21.3%)	2 (11.1%; 1.4–34.7%)	35 (15.3%; 10.9–20.6%)	32 (15.2%; 10.6 –20.7%)	2 (11.1%; 1.4–34.7%)	34 (14.9%; 10.5–20.1%)
*Haemoproteus*	4 (1.9%; 0.5–4.8%)	2 (11.1%; 1.4–34.7%)	6 (2.6%; 1.0–5.6%)	4 (1.9%; 0.5–4.8%)	2 (11.1%; 1.4–34.7%)	6 (2.6%; 1.0–5.6%)
*Leucocytozoon*	0	0	0	0	0	0
Mixed infection	0	1 (5.6%; 0.1–27.3%)	1 (0.4%; 0.0–2.4%)	0	1 (5.6%; 0.1–27.3%)	1 (0.4%; 0.0–2.4%)

### 3.2 Nested PCR and sequencing

The nested PCR assay followed by bidirectional sequencing confirmed the infection with *P. relictum* in 33 of the 34 blood samples that were tested positive by the one-step multiplex PCR. Comparison of the obtained sequences with those from the MalAvi database revealed *P. relictum* SGS1 in 29 of these birds (*Phoenicopterus roseus*: n = 24, *Alectoris graeca*: n = 1, *Lamprotornis superbus*: n = 1, *Somateria mollissima*: n = 1, *Spheniscus demersus*: n = 1, *Tetrao urogallus crassirostris*: n = 1) and *P. relictum* GRW11 in four further animals (*Phoenicopterus roseus*: n = 3, *Spheniscus demersus*: n = 1). The lineage of the *P. relictum* infecting the *Phalacrocorax carbo* could not be conclusively clarified since the nested PCR assay was negative in this case. This PCR approach allowed the identification of *Plasmodium* sp. infecting the *Alectura lathami lathami* individual as *P. elongatum* GRW06. The observed *Haemoproteus* sp. in the six individuals with monoinfection (*Bubo bubo*: n = 5, *Bubo scandiacus*: n = 1) could be specified as STRURA03. Neither a clear *Plasmodium* nor *Haemoproteus* sequence was obtained in the case of the mixed-infected *Bubo scandiacus* by this method. The occurrence of the different avian haemosporidian lineages and their prevalence are summarized in Table 1, Table 2, respectively.

### 3.3 Blood smears

No haemosporidian parasite stages were detected in the blood smears of the 237 white storks, which were sampled during the yearly ringing operation in 2019 and 2020.

### 3.4 Clinical background of haemosporidia positive individuals

At the time point of blood sampling, 19 out of 42 animals (*Phoenicopterus roseus*: n = 12, *Bubo bubo*: n = 5, *Alectura lathami lathami*: n = 1, *Phalacrocorax carbo*: n = 1) in which haemosporidian parasite DNA was detected, did not show any obvious clinical signs. Eight animals presented with minor to major health issues not directly suggestive for a haemosporidian infection, i.e., rickets, pododermatitis (*Phoenicopterus roseus*), fungal pneumonia and aerosacculitis (*Bubo scandiacus*), bacterial coelomitis, aerosacculitis, hepatitis, myo- and endocarditis (*Lamprotornis superbus)* as well as an Usutu virus infection with subsequent tubular necrosis (*Tetrao urogallus crassirostris*). Five birds (*Spheniscus demersus*: n = 2, *Somateria mollissima*: n = 1, *Bubo scandiacus*: n = 1, *Alectoris graeca*: n = 1) showed clinical signs and/or pathomorphological evidence suggestive of a haemosporidian infection. For ten further *Phoenicopterus roseus* no additional information was available. Further data about the 42 PCR positive individuals (i.e., sex, age, clinical history, postmortem findings) are provided and summarized in Table 3. The information is based on medical records on Species 360 ZIMS (Zoological Information Management Software: https://www.species360.org) of the involved zoos and on postmortem reports.

**Table 3:** Chronological data for the haemosporidia PCR positive individuals from the Zoo Basel and Berne Animal Park (underlined individuals = birds with clinical manifestations and/or pathomorphological changes suggestive for a clinical haemosporidian infection), N/A = not available.

Year	Species	Sex	Age	Clinical history	Manner of death	Postmortem findings	Haemosporidian lineage	GenBank accession number
**Zoo Basel**								
2002	*Phoenicopterus roseus*	Male	3 months	Retarded physical growth, valgus, swollen joints	Euthanasia (medical reasons)	Rickets (tibiotarsus/tarsometatarsus)	*P. relictum* SGS1	**OP710201**
								**OP727941**
2005	*Alectura lathami lathami*	Female	Juvenile	Import from USA	N/A	N/A	*P. elongatum* GRW06	**OP710234**
								**OP727976**
	*Spheniscus demersus*	Male	21.8 years	Reduced general condition, emaciation, stiff gait, bent back	Euthanasia (medical reasons)	Cachexia, arthrosis of knee and hip joints, cholangiohepatitis (multifocal), hepatic hemosiderosis	*P. relictum* SGS1	**OP710202**
								**OP727942**
2008	*Phoenicopterus roseus*	Male	Juvenile	N/A	N/A	N/A	*P. relictum* SGS1	**OP710203**
								**OP727943**
	*Phoenicopterus roseus*	Female	Juvenile	N/A	N/A	N/A	*P. relictum* SGS1	**OP710204**
								**OP727944**
	*Phoenicopterus roseus* (n = 4)	N/A	Juvenile	N/A	N/A	N/A	*P. relictum* SGS1	**OP710205**
								**OP727945**
								**OP710206**
								**OP727946**
								**OP710207**
								**OP727947**
								**OP710208**
								**OP727948**
	*Phoenicopterus roseus*	N/A	N/A	N/A	N/A	N/A	*P. relictum* SGS1	**OP710209**
								**OP727949**
2009	*Phoenicopterus roseus*	Male	3–5 months	Reduced body condition, swollen tarsal joint, malformed left tarsometatarsus, pododermatitis	Euthanasia (medical reasons)	Congenital malformation of the left tarsometatarsus, hepatitis (mild, multifocal, periportal, lymphoplasmacytic)	*P. relictum* SGS1	**OP710210**
								**OP727950**
	*Phoenicopterus roseus*	N/A	2–4 months	Ringing, implantation of microchip, wing clipping: good general and body condition	N/A	N/A	*P. relictum* GRW11	**OP710230**
								**OP727970**
	*Phoenicopterus roseus*	N/A	Juvenile	N/A	N/A	N/A	*P. relictum* SGS1	**OP710211**
								**OP727951**
	*Phoenicopterus roseus*	N/A	2–4 months	Ringing, implantation of microchip, wing clipping: good general and body condition	N/A	N/A	*P. relictum* SGS1	**OP710212**
								**OP727952**
	*Phoenicopterus roseus*	N/A	Juvenile	N/A	N/A	N/A	*P. relictum* GRW11	**OP710231**
								**OP727971**
	*Phoenicopterus roseus*	N/A	Juvenile	Ringing, implantation of microchip, wing clipping: good general and body condition	N/A	N/A	*P. relictum* SGS1	**OP710213**
								**OP727953**
	*Phoenicopterus roseus*	Female	5 months	Ringing, implantation of microchip, wing clipping: good general and body condition	N/A	N/A	*P. relictum* GRW11	**OP710232**
								**OP727972**
2010	*Phoenicopterus roseus*	N/A	N/A	N/A	N/A	N/A	*P. relictum* SGS1	**OP710214**
								**OP727954**
	*Phoenicopterus roseus* (n = 3)	N/A	2–3 months	Ringing, implantation of microchip, wing clipping: good general and body condition	N/A	N/A	*P. relictum* SGS1	**OP710215**
								**OP727955**
								**OP710216**
								**OP727956**
								**OP710217**
								**OP727957**
	*Phoenicopterus roseus* (n = 2)	N/A	5–6 months	Export to France: good general and body condition, pododermatitis	N/A	N/A	*P. relictum* SGS1	**OP710218**
								**OP727958**
								**OP710219**
								**OP727959**
	*Spheniscus demersus*	Female	26.8 years	Cloacal mass, good general and body condition	N/A	Cloacal malignant melanoma	*P. relictum* GRW11	**OP710233**
								**OP727973**
2011	*Bubo bubo*	Female	5 months	Surplus animal bred in 2011; good general and body condition, chronic ulceration at the left foot	Euthanasia (management reasons)	N/A	*Haemoproteus* sp. STRURA03	**OP710235**
								**OP727977**
	*Bubo bubo*	Female	5 months	Surplus animal bred in 2011; good general and body condition	Euthanasia (management reasons)	Pneumoconiosis	*Haemoproteus* sp. STRURA03	**OP710236**
								**OP727978**
	*Bubo bubo*	Female	Juvenile	Good general and body condition, transversal, chronic wound at the upper beak	Euthanasia (management reasons)	Pneumoconiosis	*Haemoproteus* sp. STRURA03	**OP710237**
								**OP727979**
	*Phoenicopterus roseus* (n = 2)	N/A	2–3 months	Ringing, implantation of microchip, wing clipping: good body and general condition, no pododermatitis	N/A	N/A	*P. relictum* SGS1	**OP710220**
								**OP727960**
								**OP710221**
								**OP727961**
2013	*Phoenicopterus roseus*	Male	2.5 months	Good general and body condition, pododermatitis	Euthanasia (medical reasons)	Reduced endochondral ossification and osteopenia of the femur and tibiotarsus (severe, bilateral, diffuse, chronic), tibiotarsal valgus (moderate, bilateral symmetric)	*P. relictum* SGS1	**OP710222**
								**OP727962**
2015	*Phoenicopterus roseus* (n = 3)	N/A	3–4 months	Ringing, implantation of microchip, wing clipping: good body and general condition, no pododermatitis	N/A	N/A	*P. relictum* SGS1	**OP710223**
								**OP727963**
								**OP710224**
								**OP727964**
								**OP710225**
								**OP727965**
	*Somateria mollissima*	Male	12 years	Poor general condition, moderate body condition, severe inspiratory dyspnea, pale mucous membranes, mild lameness	N/A	N/A	*P. relictum* SGS1	**OP710226**
								**OP727966**
2016	*Phalacrocorax carbo*	Female	N/A	Import from Germany	N/A	N/A	*P. relictum*	**OP727974**
2017	*Bubo scandiacus*	Female	5 years	Recurrent dyspnea due to aspergillosis since over a year; general and body condition severely reduced; poor prognosis due to lack of response to the therapy this time	Euthanasia (medical reasons)	Cachexia, pneumonia, aerosacculitis (severe, multifocal, chronic, granulomatous to necrotizing, with intralesional fungal structures consistent with *Aspergillus* sp.)	*Haemoproteus* sp. STRURA03	**OP710238**
								**OP727980**
2018	*Lamprotornis superbus*	Female	7 years	Anorexia, emaciation, reduced general condition, tachypnea	Exitus	Coelomitis, aerosacculitis, hepatitis, myo- and endocarditis (severe, multifocal to coalescing to diffuse, chronic-active, granulomatous to necrotizing, with intralesional bacteria)	*P. relictum* SGS1	**OP710227**
								**OP727967**
**Berne Animal Park**								
2018	*Tetrao urogallus crassirostris*	Male	10 years	Diarrhea, marked weight loss over four months; only animal that survived *Salmonella* outbreak; fecal sample: few *Capillaria* eggs and *Eimeria* spp. oocysts	Euthanasia (medical reasons)	Tubular necrosis (severe, diffuse, acute, with intralesional gouty tophi), hepatitis (mild, multifocal, subacute, necrotizing), proventriculitis (mild, multifocal, chronic, lymphoplasmacytic); PCR for Usutu virus (brain, kidney): Positive	*P. relictum* SGS1	**OP710228**
								**OP727968**
2019	*Bubo scandiacus*	Female	27 years	Severe, bilateral, chronic, mature cataract, unilateral lens luxation, uveitis, secondary corneal edema with superficial ulceration, insufficient plumage, unable to fly, body condition moderately reduced; poor quality of life	Euthanasia (medical reasons)	Bilateral cataract, conjunctivitis (mild, multifocal, subacute, lymphoplasmacytic), uveitis (mild, diffuse, subacute, lymphoplasmacytic), hepatitis (mild, multifocal, randomly distributed, subacute, necrotizing, heterophilic, lymphoplasmacytic)	*P. relictum, Haemoproteus* sp.	**OP727975**
								**OP727983**
2020	*Bubo bubo*	Male	9–10 months	Surplus animal bred in 2019, breeding female already laid two eggs and would have soon cast out the 2019 offspring	Euthanasia (management reasons)	N/A	*Haemoproteus* sp. STRURA03	**OP710239**
								**OP727981**
	*Bubo bubo*	Female	9–10 months	Surplus animal bred in 2019, breeding female already laid two eggs and would have soon cast out the 2019 offspring	Euthanasia (management reasons)	N/A	*Haemoproteus* sp. STRURA03	**OP710240**
								**OP727982**
	*Alectoris graeca*	Male	7 years	Severe lameness of the right leg without improvement after NSAID administration; radiographs inconspicuous	Euthanasia (medical reasons)	Nephritis, hepatitis, perineuritis (mild, multifocal, subacute, lymphoplasmacytic) with suspicion of Marek's disease, hepatic hemosiderosis	*P. relictum* SGS1	**OP710229**
								**OP727969**

## 4 Discussion

Avian haemosporidiosis is a well-known health issue in captive, non-native birds and severe courses of the disease have been reported in many zoos around the world (Fix et al., 1988; Graczyk et al., 1994a; Griner and Sheridan, 1967; Belo et al., 2009; Baron et al., 2014; Vanstreels et al., 2014; Martínez-de la Puente et al., 2015; Chagas et al., 2016; Meister et al., 2021). Zoos and animal parks often harbor a wide variety of avian species that are not native to that particular environment. Especially, migratory birds are usually infected with a biologically diverse range of avian haemosporidian species (Smith and Ramey, 2015) and can act as source of infection for suitable vectors and subsequently for avian species of zoological collections (Ejiri et al., 2009; Bueno et al., 2010; Ejiri et al., 2011; Yoshimura et al., 2014; Chagas et al., 2017; Martínez-de la Puente et al., 2020).

This study revealed a total of 83 haemosporidian sequences (multiplex PCR: n = 43, nested PCR: n = 40) from 42 host birds of ten different species. Seventy-three sequences (multiplex PCR: n = 37, nested PCR: n = 36) were detected in avian blood samples collected at the Zoo Basel and ten (multiplex PCR: n = 6, nested PCR: n = 4) at the Berne Animal Park. Since the one-step multiplex PCR provides the advantage of the detection and discrimination of the hematozoa in a single reaction with subsequent shorter reaction time, lower consumption of material and decreased risk of contamination (potential carry-over contamination of PCR products in nested protocols), the samples were first screened with this assay. Thirty-five of the sequences obtained by the multiplex PCR (Zoo Basel: n = 32, Berne Animal Park: n = 3) were identical to each other and shared a 100% BLAST identity (345/345 bp) with GenBank sequences of *P. relictum* from previously published cases of avian malaria in young Atlantic puffins (*Fratercula arctica*) from the Berne Animal Park (MT568857, MT568859, MT568860) (Meister et al., 2021). Thus, this study provides additional evidence supporting the previous suggestion that other birds in the Animal Park could have been involved as infection source for the puffins via vector transmission. Since the detected morphospecies were different in juvenile (i.e. *P. relictum* SGS1) and adult puffins (i.e. *P. matutinum* LINN1), it is more likely that the young puffins gained the infection from other zoo bird species than from the adult puffins, similarly to what was assumed for cranes in China (Jia et al., 2018). The same sequence was already reported in various avian species around the world, such as *Spheniscus demersus* from South Africa (KY653774), *Passer domesticus* (KY653772) and *Loxia curvirostra* (KY653773) from Lithuania as well as *Bubo scandiacus* (KY653754) and *Hemignathus virens* (AY733090) from the USA (Beadell and Fleischer, 2005; Pacheco et al., 2018). The obtained sequence from the Australian brush turkey (*Alectura lathami lathami*) shared 100% BLAST identity (345/345 bp) with GenBank sequences from *Plasmodium juxtanucleare* in *Myiarchus ferox* from Brazil (MG598392), *Plasmodium lutzi* in a *Diglossa lafresnayii* from Colombia (KY653815) as well as *Plasmodium elongatum* in a *Spheniscus demersus* from South Africa (KY653802) (Ferreira-Junior et al., 2018; Pacheco et al., 2018). Seven animals were infected with *Haemoproteus* sp. and the sequences obtained by the multiplex PCR shared 95.87% BLAST identity (464/484 bp) with sequences from two *Callipepla gambelii* from the USA (HQ724293, HQ724294) (Pacheco et al., 2011).

For the exact analysis of the haemosporidian lineage, the nested PCR was required and confirmed an infection with avian hematozoa in 42 of the 43 samples that were tested positive by the previous assay. Four different lineages were identified, which showed a 100% BLAST identity (478/478 bp) with already published sequences on the MalAvi database: *Plasmodium relictum* SGS1, *Plasmodium relictum* GRW11, *Plasmodium elongatum* GRW06 and *Haemoproteus* sp. STRURA03. *P. relictum* SGS1 is widespread and has already been reported in numerous avian orders (Anseriformes, Charadriiformes, Ciconiiformes, Columbiformes, Galliformes, Gruiformes, Passeriformes, Procellariiformes, Sphenisciformes, Strigiformes and Trochiliformes) in Africa, Asia, Europe, North and South America as well as Oceania. Thus, this lineage is a host generalist and it causes diseases of markedly different severity in different avian hosts (Palinauskas et al., 2008). Likewise, *P. relictum* GRW11 is relatively common (Charadriiformes, Galliformes, Passeriformes, Sphenisciformes, Strigiformes) and has been detected in Africa, Asia, and Europe. The occurrence of *Plasmodium relictum* GRW11 was already published in Switzerland in three great tits (*Parus major*) in 2013 (van Rooyen et al., 2013a, 2013b) and also in an African penguin (*Spheniscus demersus*) in Japan (Sasaki et al. unpubl: http://130.235.244.92/bcgi/malaviReport.cgi?report4=Hosts+And+Sites+Table) but this study is the first report of this lineage in a greater flamingo (*Phoenicopterus roseus*). *P. elongatum* GRW06 is also a generalist parasite and was already found in a wide variety of avian orders (Anseriformes, Apterygiformes, Ciconiiformes, Columbiformes, Coraciiformes, Falconiformes, Galbuliformes, Gruiformes, Passeriformes, Psittaciformes, Sphenisciformes and Strigiformes) in Africa, Asia, Europe, North and South America as well as Oceania. This lineage is particularly widespread in New Zealand, where it affects numerous bird species (Alley et al., 2010; Baillie and Brunton, 2011; Castro et al., 2011; Marzal et al., 2011; Ewen et al., 2012; Howe et al., 2012; Banda et al., 2013). Although sporadic deaths due to this lineage have been reported (Howe et al., 2012; Banda et al., 2013; Sijbranda et al., 2017), its pathogenicity in wild birds worldwide is generally considered to be low (Valkiūnas, 2005), which is consistent with the asymptomatic infection in the Australian brush turkey (*Alectura lathami lathami*) in this study. In Switzerland, *P. elongatum* GRW06 had not been described prior to this study and was additionally diagnosed in an Australian brush turkey (*Alectura lathami lathami*) for the first time. According to the MalAvi database, *Haemoproteus* sp. STRURA03 has only been diagnosed in Strigiformes in Europe, namely in a captive *Bubo bubo*, *Bubo scandiacus* and *Strix nebulosa* in France (Giorgiadis et al., 2020) as well as a wild *Strix uralensis* in Austria (Himmel et al. unpubl: http://130.235.244.92/bcgi/malaviReport.cgi?report4=Hosts+And+Sites+Table). This study represents the first report of *Haemoproteus* sp. STRURA03 in Switzerland.

A blood sample of a great cormorant (*Phalacrocorax carbo*) was positive for *Plasmodium* sp. in the one-step multiplex PCR; however, the nested PCR yielded negative results and the exact lineage could therefore not be conclusively clarified. The one-step multiplex PCR was repeated with freshly extracted DNA and another positive control (*P. elongatum* GRW06) to exclude contamination and a subsequent false positive result in this case. Although the nested PCR was repeated multiple times with different DNA concentrations, no positive result could be achieved. It is suspected that the DNA in this sample might have been partly degraded and that the discrepancy between the multiplex and the nested PCR might therefore be explained by the length of the PCR product (345 bp vs. 478 bp). DNA damage can result from various conditions, e.g., using very old DNA samples, using DNA extracted from formalin-fixed and paraffin-embedded samples, inefficient purifying with residual nuclease, repeated freezing and thawing, storing at room temperature, exposure to heat or physical shearing. The first three possibilities are not plausible since this blood sample was collected in 2016 and markedly older DNA could be extracted and amplified with these PCR protocols in this study, the DNA was not extracted from a FFPE tissue section, and all extraction steps were repeated. However, the remaining three causes for DNA degradation cannot be excluded. Since the sequence resulting from the one-step multiplex PCR was absolutely identical to the other *P. relictum* positive samples, *Plasmodium* sp. was specified as *P. relictum*.

*Leucocytozoon* was already detected in *Parus major* (van Rooyen et al., 2013a, 2013b), *Delichon urbicum* (van Rooyen et al., 2014) and *Hirundo rustica* (von Rönn et al., 2015) in Switzerland, but this haemosporidian species was not found in this study.

All positive individuals were captive birds and no haemosporidian DNA could be detected in free-ranging, wild individuals in this study. However, it must be mentioned that the 245 free-ranging, wild animals only represented three different avian species (*Ciconia ciconia*: n = 242; *Cygnus olor*: n = 2; *Strix aluco*: n = 1) and that 237 out of 242 tested white storks were nestlings (between six and eight weeks old) that could only be handled once during the yearly ringing operation in June/July. In an investigation about mosquito vector dynamics and infection rate of the mosquitos, 14′147 mosquitos from eight different species were caught in and around the Zoo Basel between May and September 2011, and a *Plasmodium* infection was detected in up to 7.1% of the tested mosquitos (Cereghetti, 2012). Therefore, a previous contact of the examined white storks with vectors at the time point of blood sampling was possible. However, it cannot be excluded that some of these birds might have been too young to exhibit patent infections. Although the prepatent period of avian *Plasmodium* spp. may be as short as 4–5 days, great variations among different bird species may occur and longer prepatencies were reported (Valkiūnas et al., 2018). Nevertheless, a *Plasmodium* infection was detected in several juvenile greater flamingos (*Phoenicopterus roseus*) in this study (Table 3), showing that a haemosporidian infection can also be observed in young birds. A closer contact of flamingos with the mosquitos' habitat compared to the white stork nestlings could account for earlier infections in the former avian species.

According to the MalAvi database, avian haemosporidian parasites (*Plasmodium relictum* SGS1) were only reported once in a white stork from Spain (Ferraguti et al., 2013), which might indicate that this bird species is not a common host for haemosporidia.

Due to the easier handling, small free-ranging, wild birds, particularly Passeriformes, are frequently sampled (Valkiūnas, 2005), whereas large species remain markedly neglected (Bensch et al., 2009; Clark et al., 2014) due to the difficulty to capture and obtain blood from these birds in the wild (Inumaru et al., 2017). The inclusion of the white stork samples in this study therefore provides valuable information although no haemosporidian parasites could be identified in this species by microscopic and molecular analyses. Since the blood samples of all other avian species were stored in the zoo biobanks at −80 °C, no blood smears were analyzed and the presence of gametocytes in the host blood cells could not be examined. However, this would be important to confirm that positive birds can transmit the infection. In situations where avian haemosporidia species switch hosts and evolve into new lineages, the infections are abortive and cannot be transmitted to a vector since the parasite does not fully develop in the non-adapted host due to host-parasite incompatibilities (Valkiūnas, 2011; Palinauskas et al., 2016; Valkiūnas and Iezhova, 2017). Molecular-based methods miss the identification of such infections as the PCR can detect DNA from sporozoites or extracellular merozoites, even when gametocytes are absent in blood smears (Levin et al., 2013).

From the 42 PCR positive birds, the majority did not show any obvious clinical signs, and eight animals developed clinical manifestations and or pathomorphological changes associated with etiologies other than avian malaria. Only five individuals showed clinical and pathomorphological evidence for a haemosporidian infection. In a male *Spheniscus demersus* that was euthanized due to reduced general condition and emaciation, a mixed cell hepatitis as well as hepatic hemosiderosis were histopathologically detected, which might have been related to the *Plasmodium* infection detected by the PCR. A female *Spheniscus demersus* with a previously diagnosed malignant melanoma in the cloacal area was euthanized due to reduced general condition and multiple subcutaneous swellings, which could be confirmed as metastases by the postmortem examination. Additionally, a moderately enlarged spleen and liver as well as a heterophilic to lymphoplasmacytic hepatitis were present that raised suspicion of a *Plasmodium* infection although no protozoan structures could be histopathologically detected in the affected tissues. The blood sample which was analyzed in this study and was positive for *P. relictum* SGS1 had been taken two years before the euthanasia. The male *Somateria mollissima* was reported with severely reduced general condition, marked inspiratory dyspnea and pale mucous membranes. Differential diagnoses included avian malaria, capillariosis and aspergillosis. The animal was treated using a combination of pyrimethamine and sulfadiazine although no parasites were microscopically detected by blood smear examination. Doxycycline, meloxicam, fenbendazole, toltrazuril and essential amino acids were additionally administered. No final diagnosis was made but the animal markedly improved with the initiated therapy. In the female *Bubo scandiacus* with ophthalmologic changes of the lenses, a necrotizing, heterophilic and lymphoplasmacytic hepatitis was found, which might have resulted from the ongoing mixed infection with *Haemoproteus* and *Plasmodium* sp. although no protozoans were histopathologically identified. Hepatocellular necrosis was also reported in nine great grey owls (*Strix nebulosa*) from a French zoological park (Giorgiadis et al., 2020) as well as a flamingo (*Phoeniconaias minor*) infected with *Haemoproteus* parasites (Ferrell et al., 2007). It should be noted that four (*Spheniscus demersus*: n = 2, *Somateria mollissima*: n = 1, *Bubo scandiacus*: n = 1) of the five animals with clinical signs and pathomorphological lesions belong to avian species with lacking history of co-evolution with haemosporidian parasites due to absent or very low exposure to mosquitos. It is assumed that seabirds in general may be highly susceptible to these parasites and fatal cases are relatively common. However, a protective immunity is suspected among African penguins surviving the first infection with development of low-level parasitemia without clinical manifestation (Graczyk et al., 1994b, 1995; Cranfield, 2003). This phenomenon could also explain why the blood sample of an African penguin was already PCR positive before its euthanasia two years later. In general, it must be stated that the clinical signs associated with a haemosporidian infection may be very unspecific. The same applies to the postmortem findings unless parasitic stages can be detected by histopathology.

## 5 Conclusion

This study detected haemosporidian parasites in various captive bird species from the Zoo Basel and Berne Animal Park. The infected individuals potentially act as reservoirs which in turn can lead to infection and subsequent death of particularly susceptible birds belonging to avian families such as Spheniscidae and Alcidae if management measures such as vector control and medical prophylaxis are lacking. Although all free-ranging, wild individuals were tested negative in this study, we consider the monitoring of haemosporidian infections in free-ranging, wild bird populations in and around zoological institutions an important tool to assess the risk of transmission to domestic and endangered avian species.

## Funding source declaration

This research was financially supported by the Zoologischer Garten Basel AG, Switzerland and the Institute of Parasitology, 10.13039/100009068University of Bern, Switzerland and did not receive any specific grant from funding agencies in the public, commercial, or not-for-profit sectors.

## Declaration of competing interest

The authors declare that they have no known competing financial interests or personal relationships that could have appeared to influence the work reported in this paper.
